# Comparison of laparoscopic partial nephrectomy performed with AirSeal® system vs. standard insufflator: results from a referral center

**DOI:** 10.3389/fsurg.2023.1220332

**Published:** 2023-06-27

**Authors:** Flavio Forte, Domenico Tripodi, Daniele Pironi, Emanuele Corongiu, Federica Gagliardi, Marco Frisenda, Gaetano Gallo, Antonia Quarantiello, Giuseppe Di Lorenzo, Yuri Cavaleri, Stefano Salciccia, Eleonora Lori, Salvatore Sorrenti

**Affiliations:** ^1^ Department of Urology, M.G. Vannini Hospital, Rome, Italy; ^2^ Department of Surgery, Sapienza University of Rome, Rome, Italy; ^3^ Department of Public Health and Infectious Diseases, Sapienza University of Rome, Rome, Italy; ^4^ Department of Urology, San Giovanni Battista Hospital, Foligno, Italy; ^5^ Department of Maternal-Infant and Urological Sciences, Sapienza University of Rome, Rome, Italy

**Keywords:** renal tumor, partial nephrectomy, laparoscopy, insufflation system, surgery, surgical oncology

## Abstract

**Objective:**

To compare perioperative and oncologic surgical outcomes during laparoscopic partial nephrectomy (LPN) performed by standard carbon dioxide insufflation, with those from surgeries in which the AirSeal® intelligent insufflation system was used for renal tumors.

**Materials and methods:**

A total of 27 patients with renal tumor were identified, 14 underwent LPN with AirSeal® (group A) and 13 LPN with standard insufflator (group B), respectively. Demographic baseline characteristics were similar in the two groups.

**Results:**

The size of the tumor was largest in group B (29.64 vs. 32.1 mm). The mean operative time was shorter in the AirSeal® group [group A: mean 109.0 min, median 107.5 min, interquartile range (IQR) 85; group B: mean 121.0 min, median 120.0 min, IQR 50.0]. Positive margin rates were absent in the two groups. Estimated blood loss presented a difference in the perioperative period (group A: mean 1.5 g/dL, median 1.45 g/dL; group B: mean 2.15 g/dL, median 2.2 g/dL). Time to ischemia was found to be shorter in group A with a median of 18 min compared to a median of 20 min in group B. No subcutaneous emphysema, pneumothorax, and pneumomediastinum cases occurred in either group. A postoperative complication developed in one patient requiring superselective embolization.

**Conclusion:**

In selected patients, our preliminary surgical experience has shown that the LPN procedure performed with the aid of the AirSeal® intelligent insufflation system can be used to treat even medium-/high-complexity kidney lesions, with a reduction in operating times, lower rates of complications, and perioperative blood loss.

**Clinical trial registration:**

AirSealV1.

## 1. Introduction

Renal cell carcinoma (RCC) accounts for 2%–3% of all malignancies (1), with a higher incidence in Western countries. In Europe, the mortality rate of RCC increased in the early 1990s, then stabilized, and is now decreasing (2). RCC comprises a broad spectrum of entities described in the 2016 World Health Organization (WHO) classification. There are three main histotypes of RCC: clear cell (ccRCC), papillary (pRCC: type I and type II), and chromophobe (chRCC) (3). This classification has been confirmed by genetic and cytogenetic analysis (4, 5). Surgical treatment is the choice for localized and small forms of RCC. Both in terms of choice of surgery, partial nephrectomy (PN), and radical nephrectomy (RN) and the surgical technique used (laparoscopic/robotic surgery vs. open surgery), the most appropriate approach is guided by correct clinical staging. Many retrospective studies have been performed on kidney-confined and size-limited T_1b_ RCC patient populations which have shown an overlapping cancer-specific survival between a conservative approach (PN and RN) (6, 7). In addition, studies have demonstrated improved preservation of renal function in patients undergoing PN, with a decrease in metabolic and cardiologic disorders (8, 9) and a decrease in deaths due to cardiac issues with an increase in overall survival (8–12). Therefore, in addition to oncologic radicality, the primary goal for an ideal PN is maximum preservation of renal function (13). Even in patients with preoperative renal failure, it is advisable to lean toward a more conservative approach to limit the long-term risks of needing hemodialysis treatment. In the international literature, emphasis has been placed on the role played, to the detriment of renal function, by renal ischemia time and renal parenchyma loss during PN surgery. However, on the other hand, no significant differences in terms of days of hospitalization, peri- and postoperative complications, number of blood transfusions, and estimated blood loss (EBL) were noted between PN and RN. Considering these data, it is important to reduce or eliminate the ischemia time and remove healthy renal parenchyma during mass resection or rendered nonfunctional by subsequent hemostatic suturing. Moreover, laparoscopic surgery has gained acceptance in the treatment of urologic oncologic disease with less postoperative pain, less blood loss, and shorter hospital stay than open surgery (14). Complications that can occur following carbon dioxide (CO_2_) insufflation during laparoscopic surgery include subcutaneous emphysema (SCE), pneumothorax (PTX), and pneumomediastinum (PMS). Homeostasis can be negatively affected by the increase in intra-abdominal pressure, consequent to the insufflation of CO_2_, causing significant changes in the cardiovascular and respiratory systems (15, 16). Conventional CO_2_ insufflation systems frequently have an inadequate response, generally due to a delay, to intraoperative pressure loss due to aspiration or smoke evacuation. A valveless insufflation system introduced in 2011, now commercially available as AirSeal® Insufflation System (AIS) (Conmed, Utica, NY, USA), has significant advantages over conventional insufflation systems. The AirSeal® mode is designed to provide CO_2_ insufflation that ensures stable pneumoperitoneum and continuous suction of surgical vapors during laparoscopic procedures. This feature is very useful during procedures where numerous monopolar instrumentations are used. The objective of our study was to compare the surgical outcomes of laparoscopic partial nephrectomy (LPN) procedures performed using the standard CO_2_ insufflation with those of procedures in which the AirSeal® intelligent insufflation system was used.

## 2. Materials and methods

### 2.1. Methods

**Figure 1:**
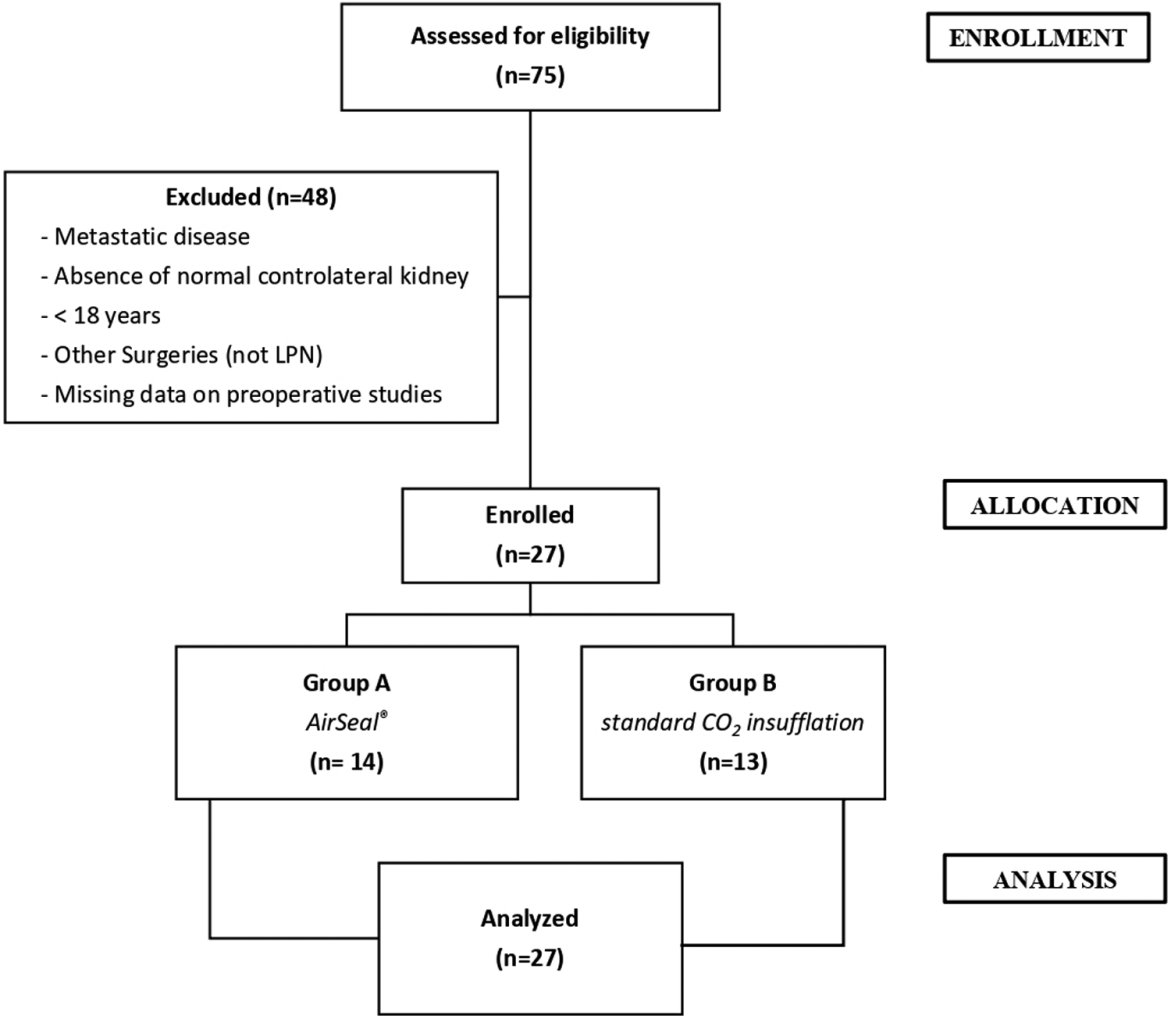
Patients’ enrollment process. LPN, laparoscopic partial nephrectomy.

Data were extracted from a maintained renal tumor database approved by our institutional review board. All the patients underwent preoperative imaging examinations using contrast-enhanced computed tomography (CT). Between January 2019 and December 2019, 27 patients with localized RCC, treated with LPN, were enrolled. Exclusion criteria were, age under 18 years, metastatic disease at presentation, absence of a normal contralateral kidney (including bilateral disease), and missing data on preoperative studies. The patients’ enrollment process was shown in Figure 1. Each patient provided consent for inclusion in our institution's database, in which we noted their medical history, clinical data, postoperative follow-up, and any complications. Comorbidities were assessed using the Charlson Comorbidity Index (CCI) score (17) and the American Association of Anesthesiologist (ASA) score (18). Tumor complexity was evaluated using the RENAL (radius, exophytic/endophytic, nearness, anterior/posterior, location) nephrometry scoring system on preoperative imaging and was stratified as low, moderate, or high, if the RENAL score was 4–6, 7–9, and 10–12, respectively (19). All LPN surgical procedures were performed by experienced and high-volume surgeon. The study was conducted in accordance with the principles of the Declaration of Helsinki and the guidelines for good clinical practice, and written informed consent was obtained from all included patients. Surgical outcomes, including operative time, EBL, transfusion rate, positive surgical margin, and complications (including conversions), were compared between the two groups. The first group (group A, 14 patients) was operated on using the AirSeal® system at 12 mmHg, while the second group (group B, 13 patients) was operated on using the standard CO_2_ insufflation at 12 mmHg. Anatomopathological evaluation of the treated renal lesions was performed by a pathologist with experience in urological surgery, using the latest criteria (2016) of the WHO (15) and those of the Fuhrman classification (20). “Positive” surgical margins were defined by the presence of tumor cells at the excision surface of the parenchyma. To assess postoperative pain, a validated questionnaire (general/shoulder) was used; additionally, a chest x-ray read by a radiologist was routinely performed to identify PMS, PTX, and SCE. Complications were classified and recorded based on the modified Clavien–Dindo classification (21).

### 2.2. Statistical analysis

Descriptive analysis was performed using frequencies and percentages for categorical variables and means, standard deviations, medians, and interquartile ranges (IQR) for continuous variables. The latter were also tested for normality using the Shapiro–Wilk test. Continuous and categorical variables were compared using the Mann–Whitney test and Fisher's exact test or chi-square test, respectively. Second, multivariate logistic regression analysis was performed to build three regression models using three different outcomes. To evaluate the operative time and use it in a regression model, the first regression model was used to test the outcome of quick surgery by taking the lower quartile of the operative time and using it as a cutoff for a binary variable which distinguishes fast operations from average or slow operations (0 ≥ 79 min; 1 ≤ 78 min). A second regression model was constructed to test the outcomes of ischemia. To evaluate the presence of ischemia during surgery, a binary variable was created using the variable warm ischemia time, considering patients with no ischemia time as patients without ischemia. On the other hand, every value of >1 was considered a patient with ischemia. A third regression model was built to test for the occurrence of transfusions. Univariate analysis was performed out to identify all possible covariates to be included in the models. Variables with *p *< 0.20 were included in the multivariate models. Subsequently, the results were expressed as adjusted odds ratios (ORs), 95% confidence intervals (CI), and *p*-values. Final models were selected by backward elimination of non-significant variables based on the likelihood-ratio test (cutoff *p*-value = 0.05). In the final multivariate regression models, results with *p* < 0.05 were considered significant. The following variables were tested: use of AirSeal® technique (0 = no; 1 = yes), portion of kidney treated surgically (0 = inferior polar region, 1 = medial polar region, 2 = superior polar region), blood transfusion after surgery (0 = no; 1 = yes), type of surgical approach (0 = enucleation; 1 = other type of surgeries), ASA index (0 = low risk; 1 = medium and high risk), changes of hemoglobin values before and after surgery, the time needed for the surgery, the size of the lesion, and RENAL score. All analyses were performed using Stata v. 17 software (Stata Corporation, College Station, TX, USA). All data were anonymously processed.

## 3. Results

The two groups of patients had similar preoperative characteristics. The mean age was 61.3 years (median 60; IQR 19.0) for group A and 65.9 years (median 66; IQR 15.0) for group B, respectively. The mean lesion size, as assessed by contrast-enhanced CT, was 29.6 mm (median 28.5 mm; IQR 20.0) and 32.1 mm (median 27 mm; IQR 23.0), respectively. The observed RENAL nephrometry score (8) was on average 6.2 (median 6.5; IQR 2.0) for group A, while for group B, it was 5.0 (median 5.0; IQR 2.0), indicating a dissimilar complexity of the treated tumors that influenced the choice of the most appropriate surgical approach. Other patient characteristics are summarized in Table 1. The mean operative time was shorter in the first group (group A: mean 109.0 min, median 107.5 min, IQR 85; group B: mean 121.0 min, median 120.0 min, IQR 50.0). Regarding the “hot” ischemia time, it too was found to be shorter in group A with a median of 18 min, compared with a median of 20 min in group B. In addition, more cases performed as “zero ischemia” was observed in group B (11 vs. 8). No positive surgical margins were evident in either group (Table 2). No SCE cases occurred in either group. There was a conversion to open surgery required to complete the procedure in only one patient of group B. A major postoperative complication developed in one patient in group B (class III according to the Clavien–Dindo classification): renal bleeding that required superselective embolization in interventional radiology (Table 3).

**Table 1:** Baseline characteristics.

Variable	Group A (AirSeal®)	Group B (standard insufflation)
Number of patients	14	13
Age (years)		
Mean	61.3	65.9
Median	60	66
IQR	19	15
Tumor size (mm)		
Mean	29.6	32.1
Median	28.5	27.0
IQR	20	23
RENAL score		
Mean	6.2	5.0
Median	6.5	5.0
IQR	2	2
Sex	*n* (%)	*n* (%)
Male	11 (78)	9 (70)
Female	3 (22)	4 (30)
Laterality		
Right	6 (42.8)	7 (53.8)
Left	8 (57.1)	6 (46.1)
Location		
Anterior	1 (7.1)	2 (7.4)
Posterior	6 (42.8)	3 (11.1)
Hilar	0 (0)	0 (0)
Neither	7 (50.0)	8 (29.6)

IQR, interquartile range; RENAL, radius, exophytic/endophytic, nearness, anterior/posterior, location.

**Table 2:** Effects of insufflation type on operative time, ischemia time, and blood loss.

Variable	Group A (*n* = 14)	Group B (*n* = 13)
Operative time (min)		
Mean	109	121
Median	107.5	120
IQR	85	50
Warm ischemia time (min)		
Mean	18	19
Median	18	20
IQR	2	6
Estimated blood loss—delta Hb (g/dL)		
Mean	1.5	2.15
Median	1.45	2.2
IQR	0.8	1.6
** **	*n* (%)	*n* (%)
Zero ischemia	8 (57.1)	11 (84.6)
Positive surgical margins	0 (0)	0 (0)

Group A, AirSeal®; group B, standard CO_2_ insufflation; IQR, interquartile range; Hb, hemoglobin.

**Table 3:** Rates of peri- and postoperative complications.

Variable	Group A (*n* = 14)	Group B (*n* = 13)
	*n* (%)	*n* (%)
Postoperative blood transfusion	0 (0)	1 (7.7)
Conversion to open	0 (0)	1 (7.7)
Cases of SCE	0 (0)	0 (0)
Development of postoperative acute kidney injury	0 (0)	0 (0)

Group A, AirSeal®; group B, standard CO_2_ insufflation; SCE, subcutaneous emphysema.

## 4. Discussion

In some studies, warm ischemia lasting longer than 25 min has been shown to determine irreversible renal injury after PN (22). However, recent work shows that every minute counts if the renal hilum is clamped during PN (23). As a result, minimally invasive approach is particularly challenging because advanced surgical skills are needed to achieve effective tumor resection, maintain hemostasis, and perform subsequent renorrhaphy, within the shortest time possible. The ability to clearly see the surgical field is also crucial to facilitating surgical maneuvers and reducing overall operative time. By delaying the intraoperative pressure drop, the conventional CO_2_ insufflation systems often respond. In fact, conventional insufflators typically switch from CO_2_ insufflation for approximately 3 s to a pause of 1 s and then measure the pressure and cyclically reinflate to maintain the set pressure. Therefore, conventional mechanical insufflators cause cyclic oscillation of the pressure inside the abdomen (24); as a consequence, these fluctuations, suction maneuvers, or smoke evacuation usually lead to the collapse of the abdominal cavity, which can only be avoided and compensated by increasing the gas insufflation pressure. Postoperative shoulder pain can occur due to excessive stretching of the diaphragm muscle fibers caused by increased CO_2_ pressure (25). Conventional trocars have a cannula with a proximal unidirectional valve and a cannula with a distal hollow thread. Gas escapes from the abdominal cavity when the trocar valves are opened to accommodate the instruments. The resulting moisture and surgical smoke impair the surgeon's vision and often contaminate laparoscopic lenses, requiring suctioning and cleaning of the instruments, which prolongs operative time (26). AirSeal® therefore represents a new insufflation system that uses trocars without valves or membranes, responding immediately to slight variations in intra-abdominal pressure (27). The AirSeal® system reduces the consumption of CO_2_ during surgery (28). Thanks to real-time pressure equalization, the AirSeal® system allows the surgeon to easily work at lower pressures, reaching up to 7 mmHg, with inlet gas flow never exceeding 3 L/min, providing further benefits to the patient, who finds relief both during the procedure and during postoperative recovery. To date, few studies have examined the role of the AirSeal® system compared with the standard CO_2_ insufflation for the same type of surgery. Herati et al. (27) reported the first prospective comparative study between the AirSeal® system (26 patients) and a standard trocar (25 patients). The authors find that the mean operative time as well as the amount of CO_2_ consumed were significantly lower in the group where the AirSeal® system was used. Annino et al. (29) published a comparative study between the AirSeal® system (67 patients) and the standard insufflation system (55 patients) in the field of robotic partial nephrectomy. The mean operative and warm ischemia time was significantly shorter in the first group. Feng et al. (30) and Desroches et al. (31) also compared the standard CO_2_ insufflation system with the valveless system used in robotic PN in a prospective randomized trial. The results of our study suggest that the average operative times and, consequently, patient CO_2_ exposure and potential adverse outcomes were shorter in the group of patients operated on with the AirSeal® system; when the “zero ischemia” procedure was not feasible, the use of the intelligent insufflation system allowed for less bleeding, especially during the continuous suction phases of tumor resection, improving the visibility of the surgical resection margins and arterial trunk afferents to the tumor, thus selectively controlling them. All of this allowed for late arterial clamping, followed by early unclamping, significantly reducing the “hot ischemia” time. Another advantage is that the removal of parts of the anatomical tissue or the application of gauze and/or hemostatic sponges does not cause any dysfunction of the trocars, since they do not have valves that can fail, as is usually the case with standard trocars, improving the visualization and efficiency of surgical maneuvers, resulting in a reduction in surgical time. In addition, because the valveless trocar system functions at low flow, its use has the potential to reduce cardiopulmonary system compromise from CO_2_ insufflation; therefore, the benefits of these trocars can be significant, especially in cases requiring longer operative times or in patients with severe chronic cardiopulmonary disease. AirSeal® was a significant predictor of a shorter operative time (OR 41.09; 95% CI 1.07–1,566.0) without any other influencing factors. Regarding ischemia, a significant negative association has been found with surgery in the polar medial region of the kidney (OR 0.003; 95% CI 9.22e−06–0.98). Furthermore, a longer intervention time was associated with an increased risk of ischemia (OR 1.06; 95% CI 1.00–1.13) (Table 4). There was no significant association with blood transfusion between groups. The preliminary results of our study seem to align with the previous experiences of other authors (Table 5), highlighting that this system can be used to treat even medium-/high-complexity renal lesions, without compromising oncological results. The main limitation of our study is the small sample size. Moreover, although the data were prospectively collected, the analysis was retrospective and, therefore, subject to the inherent limitations of retrospective analyses. Another limitation is the absent secondary analysis of transperitoneal vs. retroperitoneal approach for which we were not warrant.

**Table 4:** Multivariate logistic regression.

Variable name	Model 1 predictors of quick surgery			Model 2 predictors of ischemia			Model 3 predictors of transfusion		
	OR	pV	95% CI	OR	pV	95% CI	OR	pV	95% CI
AirSeal®	41.09	**0** **.** **04**	1.07–1,566.0	141.24	0.08	0.47–417.0	11.71	0.43	0.02–5,272.0
Location									
Inferior polar region	Ref.	–	–	Ref.	–	–	Ref.	–	–
Medial polar region	0.04	0.19	<0.01–4.85	0.03	**0** **.** **05**	<0.01–0.98	4.88	0.51	0.04–564.0
Superior polar region	0.19	0.32	<0.01–5.25	0.01	0.08	<0.01–1.74	1.16	0.93	0.02–49.7
Blood transfusion									
No	Ref.	–	–						
Yes	3.27	0.50	0.09–110.0						
Surgical procedure									
Enucleation	Ref.	–	–						
Others	0.08	0.12	<0.01–1.9						
ASA index									
Medium–High	Ref.	–	–						
Low	1.80	0.73	0.06–53.7						
Delta Hb				4.05	0.32	0.44–67.2	1.52	0.61	0.30–7.78
Surgical time				1.06	**0** **.** **04**	1.0–1.13	1.05	0.12	0.99–1.11
Renal score				5.18	0.09	0.75–35.8	0.32	0.31	0.03–2.95
Size				0.78	0.12	0.58–1.06			

ASA, American Society of Anesthesiologists; RENAL, radius, exophytic/endophytic, nearness, anterior/posterior, location; Hb, hemoglobin.

Indicated by bold values Delta HB - estimated blood loss.

**Table 5:** Comparative analysis with previous studies.

Comparative data	Present study	Annino et al. (29)	Feng et al. (30)	Desroches B et al. (31)
	Median	Median	Median	Median
Age (years)	59, 5	66, 3	60, 2	60, 1
Tumor size (mm)	27	40	NA	NA
RENAL score	6	6	NA	NA
Operative time (min)	120	140	180	NA
Warm ischemia time ( min)	18	11	NA	NA
Blood loss—delta Hb (g/dL)	1.5	1.9	NA	NA
Sex	*n*	*n*	*n*	*n*
Male	20	47	37	129
Female	7	20	25	72
AirSeal® population	14	67	62	66 (*P* = 12 mm Hg) 69 (*P* = 15mm Hg)
Positive surgical margins, nr	0	3	NA	NA
Cases of SCE, nr AirSeal® population	0	0	6 (*P* = 12 mm Hg)12 (*P* = 15mm Hg)	9 (*P* = 12 mm Hg) 21 (*P* = 15mm Hg)

SCE, subcutaneous emphysema; RENAL, Radius, Exophytic/Endophytic, Nearness, Anterior/Posterior, Location; Hb, hemoglobin.

## 5. Conclusion

Although not comparable with studies with a larger number of patients undergoing minimally invasive PN surgery, our preliminary experience has shown that the LPN procedure performed with the aid of the AirSeal® intelligent insufflation system can be used to treat even medium-/high-complexity renal lesions, with a reduction in operating time, “warm ischemia” time, and perioperative blood loss. However, the uniqueness of our study is represented by the fact that for the first time, the advantages of this system were investigated only in the field of laparoscopic partial nephrectomy in renal cell carcinoma. Furthermore, our data investigated the feasibility and safety of an LPN approach using a smart insufflation system.

## Data Availability

The raw data supporting the conclusions of this article will be made available by the authors, without undue reservation.
